# Pharmacological postconditioning with Neuregulin-1 mimics the cardioprotective effects of ischaemic postconditioning via ErbB4-dependent activation of reperfusion injury salvage kinase pathway

**DOI:** 10.1186/s10020-018-0040-7

**Published:** 2018-07-31

**Authors:** Fuhua Wang, Huan Wang, Xuejing Liu, Haiyi Yu, Bo Zuo, Zhu Song, Ning Wang, Wei Huang, Guisong Wang

**Affiliations:** 1 Department of Cardiology, Peking University Third Hospital, Key Laboratory of Cardiovascular Molecular Biology and Regulatory Peptides, Ministry of Health, Key Laboratory of Molecular Cardiovascular Sciences, Ministry of Education. Beijing Key Laboratory of Cardiovascular Receptors Research, 9, HuaYuanBei Road, HaiDian District, Beijing, 100191 People’s Republic of China; 2 Institute of Cardiovascular Sciences and Key Laboratory of Molecular Cardiovascular Sciences, Ministry of Education, Peking University Health Science Center, 38, XueYuan Road, HaiDian District, Beijing, 100191 People’s Republic of China

**Keywords:** Neuregulin-1, Pharmacological postconditioning, Ischaemic postconditioning, Myocardial reperfusion injury, ErbB4

## Abstract

**Background:**

The protective effect of Neuregulin-1 (NRG-1) on heart failure is well established. In this study, we assessed whether NRG-1 could protect the heart by mimicking the cardioprotective effects of ischaemic postconditioning (IP).

**Methods:**

We used a myocardial reperfusion injury rat model in vivo to compare the cardioprotective effects of NRG-1(3 μg/kg, iv. at the onset of reperfusion) and IP. In Langendorff isolated heart perfusion experiments, we used the erythroblastic leukaemia viral oncogene homolog 4 (ErbB4) inhibitor AG1478, a phosphatidylinositol 3-kinase (PI3K) inhibitor LY294002 and a mitogen-activated protein/extracellular signal regulated kinase (MEK) inhibitor PD98059 to clarify whether the protective effects of NRG-1and IP depend on the NRG-1/ErbB4 signals and the reperfusion injury salvage kinase (RISK) pathway. Infarct size was detected by Evans blue and TTC. Apoptosis was detected by TUNEL assays. The expression of NRG-1/ErbB4 and downstream ERK1/2, AKT, AMPK and p70s6K were detected by western blotting. Hematoxylin/eosin (H&E) staining was used for histological analysis.

**Results:**

We found that NRG-1 and IP had similar effects on reducing myocardial infarct size and apoptosis in vivo. NRG-1 heart protein levels were upregulated in the IP group. Phosphorylation of AKT, ERK1/2 and ErbB4 were also increased in both the IP and NRG-1 groups. Furthermore, in Langendorff analyses, the ErbB4 inhibitor AG1478 suppressed the phosphorylation of ErbB4 and the RISK pathway and aggravated myocardial edema and fiber fracture, thereby inhibited the cardioprotective effects in both the IP and NRG-1 groups. For assessment of downstream signals, the PI3K inhibitor LY294002 and the MEK inhibitor PD98059 suppressed the phosphorylation of AKT and ERK1/2 respectively and abolished the cardioprotective effects induced by IP and NRG-1.

**Conclusion:**

In conclusion, both IP and NRG-1 could reduce infarct size and apoptosis through ErbB4-dependent activation of the RISK pathway in the same model; these results indicated the therapeutic potential of NRG-1 as a pharmacological postconditioning agent against myocardial reperfusion injury.

## Background

Acute myocardial infarction (AMI) due to coronary artery occlusion is one of the most common and severe heart diseases in the world (Mozaffarian et al., 2015). Reperfusion of ischaemic myocardium is crucial for salvaging the heart tissue. However, restoration of blood flow could lead to myocardial ischaemia-reperfusion (IR) injury (Ibanez et al., 2015). Ischaemic postconditioning (IP), defined as brief episodes of IR at the onset of reperfusion, is a common strategy to salvage the myocardium suffering from reperfusion injury (Heusch, 2015). The cardioprotective effects of IP rely on activation of the reperfusion injury salvage kinase (RISK) pathway (Hausenloy & Yellon, 2004), which involves phosphatidylinositol 3-kinase/AKT (PI3K/AKT) (Tsang et al., 2004) and extracellular signal-regulated kinase 1/2 (ERK1/2) signalling (Yang et al., 2004). However, IP application is limited to patients with AMI undergoing percutaneous coronary intervention. Therefore, pharmacological approaches that can mimic the cardioprotective effects induced by IP will be more accessible and benefit more patients. Previous studies showed several substances, such as adenosine, bradykinin and opioids, could act as pharmacological postconditioning agents to protect hearts from IR injury. Three major intracellular signal transduction pathways are involved in this protective effects: the eNOS/PKG pathway, the RISK pathway and the survivor activating factor enhancement pathway. However, the cardioprotective effects of these protective factors in clinical settings still remain controversial (Kleinbongard & Heusch, 2015). Therefore, it is still of importance to find more potential cardioprotective factors as pharmacological postconditioning agents.

Neuregulin-1 (NRG-1), a member of the epidermal growth factor (EGF) family (Parodi & Kuhn, 2014), has been shown to play a critical role in the regulation of cardiac development (Odiete et al., 2012) and adult cardiac function (Sawyer & Caggiano, 2011). NRG-1 binds to erythroblastic leukaemia viral oncogene homolog 4(ErbB4), a tyrosine kinase receptor, and induces the conformational changes in ErbB4 that allows dimerization with ErbB2 or ligand-activated ErbB4. These changes provided docking sites for downstream signals that mediates several processes in cardiomyocytes (Odiete et al., 2012). Many studies have shown the potential effects of NRG-1 on heart failure. Administration of NRG-1 improved cardiac function via SERCA2a and cMLCK in a rat heart failure model (Gu et al., 2010). A phase II clinical trial demonstrated that NRG-1 significantly enhanced cardiac function in patients with chronic heart failure (Gao, 2010). NRG-1 also suppressed apoptosis in injured primary cardiac myocytes through RISK pathway activation (Xu et al., 2014; Jie et al., 2012; Fukazawa, 2003; Liu et al., 2005). In animal experiments, the infarct area induced by IR was increased when NRG-1 was specifically knocked out in the microvascular endothelial cells of mice. This study showed that NRG-1 has an important role against myocardial reperfusion injury in the heart (Hedhli et al., 2011). A recent report demonstrated that exogenous NRG-1 could reduce the infarct size (IS) in a myocardial reperfusion injury in situ model (Ebner et al., 2015). No study to date has detected protective effects of NRG-1 on IR as pharmacological postconditioning agent in a rat model, and comparison of the cardioprotective effects of NRG-1 and IP has not yet performed in the same experimental model. The protective effects of NRG-1 and IP against myocardial reperfusion injury are mediated by the same downstream signalling RISK pathway. Thus, in this study, we compared the protective effects of NRG-1 and IP on IR and also investigated the possible effects of the NRG-1/ErbB signalling pathway on IP. We found that NRG-1 had a similar protective effect in reducing IS compared with IP, and both NRG-1 and IP could reduce apoptosis through ErbB4-dependent activation of the RISK pathway in rat in vivo and the Langendorff model of myocardial reperfusion injury. These findings indicate the therapeutic potential of NRG-1 as a pharmacological postconditioning agent against myocardial reperfusion injury.

## Methods

### Animals

Male adult Sprague-Dawley rats weighing 200–300 g were purchased from the Laboratory Animal Center of Peking University. The Principles of Laboratory Animal Care (NIH publication no. 85–23, revised 1996) were followed, and the experimental protocol was approved by the Animal Care Committee, Peking University Health Science Center.

### Analysis of myocardial reperfusion injury in vivo

#### Myocardial reperfusion injury model

Rats were anaesthetised by sodium pentobarbital (50 mg/kg) through intraperitoneal injection and then ventilated with a rodent respirator (ALCV9A; Shanghai Alcott Biotech Co., Ltd., Shanghai, China) after intubation. A left thoracotomy was performed to open the thorax through the fourth or fifth intercostal space, and the heart was exposed after the ribs were gently distracted. After the pericardium was removed, a 6–0 silk suture was placed under the left anterior descending coronary artery (LAD), and before the suture was tightened, two suture loops were put through the two ends of the suture to reocclude LAD after the ligation. The coronary artery was occluded for 45 min. Ischaemia was confirmed by blanching of the myocardium, dyskinesia of the ischaemic region and ST segment elevation on the ECG. Then, the heart was reperfused for 24 h by loosening the knot, and this was confirmed by a marked hyperemic response at reperfusion (Tamareille et al., 2009).

#### In vivo experimental protocol

The rats were randomly divided into four groups: (1) CON (control) group, in which the heart was exposed, but the LAD was not occluded; (2) IR (ischaemia reperfusion) group, in which the LAD was occluded for 45 min and reperfused for 24 h; (3) IP (ischaemic postconditioning) group, in which the LAD was occluded for 45 min, and at the onset of reperfusion, intervention of 6 cycles of 30-s occlusion/30-s reperfusion was performed as the postconditioning treatment, and then the heart was reperfused for 24 h; (4) NRG-1 (IR + NRG-1) group, in which the LAD was occluded for 45 min, and just before reperfusion, recombinant human NRG-1β2 (3 μg/kg, Prospec, Israel) was intravenously injected via the jugular vein, and the heart was reperfused for 24 h. The dosage of NRG-1 used in this study was chosen based on a previous study (Fang et al., 2010). In the present study, we reperfused the heart for 24 h to observe clear infarct demarcation as shown in previous studies (Liu et al., 2014; King et al., 2014).

### Myocardial reperfusion injury model in Langendorff isolated heart

#### Heart preparation

As previously described (Bell et al., 2011), rats were anaesthetised by sodium pentobarbital (50 mg/kg), and the heart was removed to a Langendorff apparatus and retrogradely perfused through the aorta with the Krebs-Henseleit (K-H) buffer (NaCl 118.5 mM, NaHCO_3_ 25.0 mM, KCl 4.7 mM, MgSO_4_ 1.2 mM, KH_2_PO_4_ 1.2 mM, glucose 11 mM and CaCl_2_ 2.5 mM, at pH 7.4 and gassed with 95% O_2_ and 5% CO_2_ at 37 °C). With a constant pressure of 70 mmHg, the heart was equilibrated for 20 min.

#### Isolated heart experimental protocol

The rats were randomly assigned to six groups: (1) CON (no-intervention) group; (2) IR group, in which the coronary flow was stopped for 30 min, and then, the heart was reperfused; (3) IP group, in which 6 cycles of 10-s occlusion/10-s reperfusion were performed at the onset of reperfusion, and then the heart was reperfused; (4) IP + AG1478 (an inhibitor of ErbB4) group, in which the same treatment as the IP group was performed, except 2 μM AG1478 was perfused for 10 min before the reperfusion and lasted for 20 min at the reperfusion period (the dosage of AG1478 was determined according to a previous study (Cai et al., 2016)); (5) NRG-1 group, at the onset of reperfusion, 20 ng/ml NRG-1 was perfused for 20 min (the dosage of NRG-1 was determined according to a previous study (Ebner et al., 2015)); (6) NRG-1 + AG1478 group, in which the treatment was the same as the NRG-1 group, except 2 μM AG1478 was perfused for 10 min before the reperfusion and lasted for 20 min at the reperfusion period. The heart was reperfused for 2 h for TTC staining or 20 min for western blotting.

#### Isolated heart experimental protocol-2

The rats were randomly assigned to eight groups: (1) CON (no-intervention) group; (2) IR group, in which the coronary flow was stopped for 30 min, and then, the heart was reperfused; (3) IP group, in which 6 cycles of 10-s occlusion/10-s reperfusion were performed at the onset of reperfusion, and then the heart was reperfused; (4) IP + LY294002 (LY, an inhibitor of PI3K) group, which underwent similar treatment as the IP group, except 20 μM LY294002 was perfused for 10 min before the reperfusion and lasted for 20 min at the reperfusion period (the dosage of LY294002 was determined according to a previous study (Tamareille et al., 2009)); (5) IP + PD98059 (PD, an inhibitor of MEK) group, which underwent the same treatment as the IP group, except 20 μM PD98059 was perfused for 10 min before the reperfusion and lasted for 20 min at the reperfusion period (the dosage of PD98059 was determined according to a previous study (Tamareille et al., 2009)); (6) NRG-1 group, at the onset of reperfusion, 20 ng/ml NRG-1 was perfused for 20 min; (7) NRG-1 + LY294002 group, which underwent the same treatment as the NRG-1 group, except 20 μM LY294002 was perfused for 10 min before the reperfusion and lasted for 20 min at the reperfusion period; (8) NRG-1 + PD98059 group, which underwent the same treatment as the NRG-1 group, except 20 μM PD98059 was perfused for 10 min before the reperfusion and lasted for 20 min at the reperfusion period. The heart was reperfused for 2 h for TTC staining or 20 min for western blotting.

### Assessment of IS

The staining was performed as previously described (Xie et al., 2015). At the end of the reperfusion, LAD was ligated again, and the heart was retrogradely infused with Evans blue (Sigma, St. Louis, MO, USA, 0.25% in saline) from the aorta. The non-ischaemic area was stained blue, indicating the area at risk (AR, non-blue region). Then, the heart was frozen at − 20 °C for 30 min and sectioned into 6 slices of 2 mm thickness. The slices were incubated in 1% triphenyltetrazolium chloride (TTC, Sigma, St, Louis, MO, USA) in phosphate buffer (pH 7.4) for 10 min at 37 °C and subsequently soaked in 4% paraformaldehyde for 24 h. TTC staining could differentiate the IS (white region) from the non-infarct AR (red region). In the Langendorff perfusion experiment, rat heart was only stained by TTC without the infusion of Evans blue as described previously (Bell et al., 2011). Non-infarct area of the left ventricle (LV) was stained red. Finally, the slices were arranged from apex to base and digitally photographed. Digital images of the slices were analysed by ImageJ software (NIH, USA) to measure the IS. The final result is expressed as IS/AR% for in vivo results and IS/LV% for Langendorff experiments as previously described (Tamareille et al., 2009).

### Western blotting

At the end of the reperfusion, the LV in the risk area was freeze-clamped in liquid nitrogen before being stored at − 80 °C. Frozen tissue samples were homogenised in RIPA solution, and 80 μg extracted protein was subjected to western blotting. SDS-PAGE and immunoblotting were performed as previously described (Xu et al., 2015). ECL chemiluminescence was used for detection of the bands by an imaging system (molecular imager, ChemiDoc XRS, Bio-Rad, USA), and the densities of the bands were also determined semi-quantitatively using the same system. All protein levels were normalized to that of GAPDH. Primary antibodies used in the experiment were as follows: ^202^Thr/^204^Tyr-P-ERK1/2, T-ERK1/2, ^473^Ser-P-AKT, T-AKT (pan),^389^Thr-P-p70S6k, p70S6k, ^172^Thr-P-AMPK, AMPK (rabbit monoclonal antibodies, Cell Signaling Technology, USA) and caspase 3 antibody (rabbit polyclonal antibody, Cell Signaling Technology, USA); ^1248^Tyr-P-ErbB2, ErbB2, ^1284^ Tyr-P-ErbB4, T-ErbB4, and NRG-1 (rabbit polyclonal antibodies, Abcam, USA); GAPDH (mouse anti-human monoclonal antibody, Millipore, USA) and appropriate horseradish peroxidase-conjugated secondary antibody (ZSGB-BIO, China).

### Evaluation of apoptosis

Terminal deoxynucleotidyl transferase-mediated dUTP nick end labelling (TUNEL) assays were performed to detect apoptotic cells of the heart tissue sections in the risk area by an in situ cell death detection kit (Roche Applied Science, USA) according to the manufacture’s instruction. The nuclei were counted in 10 random fields of an optical microscope (400X, Leica, Germany) for each section, and the results are expressed as a percentage of TUNEL-positive nuclei in the total cell nuclei.

### Histological analysis

After 2 h perfusion, the ischaemic myocardial tissue of the isolated heart was fixed in 4% polyformaldehyde formalin. The paraffin-embedded sections were stained with hematoxylin/eosin (H&E) according to a previous study (Xu et al., 2015). Then the tissue sections were evaluated for histological changes by an optical microscope (400X, Leica, Germany).

### Statistical analysis

All data are presented as the mean ± SEM. Statistical comparisons between the groups were performed using one-way ANOVA followed by Neuman-Keuls post-hoc test or followed by Mann-Whitney test for non-parametric data. The statistical analyses were performed by GraphPad Prism 5.0 (Graph Pad Software, San Diego, CA). A value of *p* ≤ 0.05 was considered significant.

## Results

### The equivalent cardioprotective effects of IP and NRG-1 in vivo

**Fig. 1:**
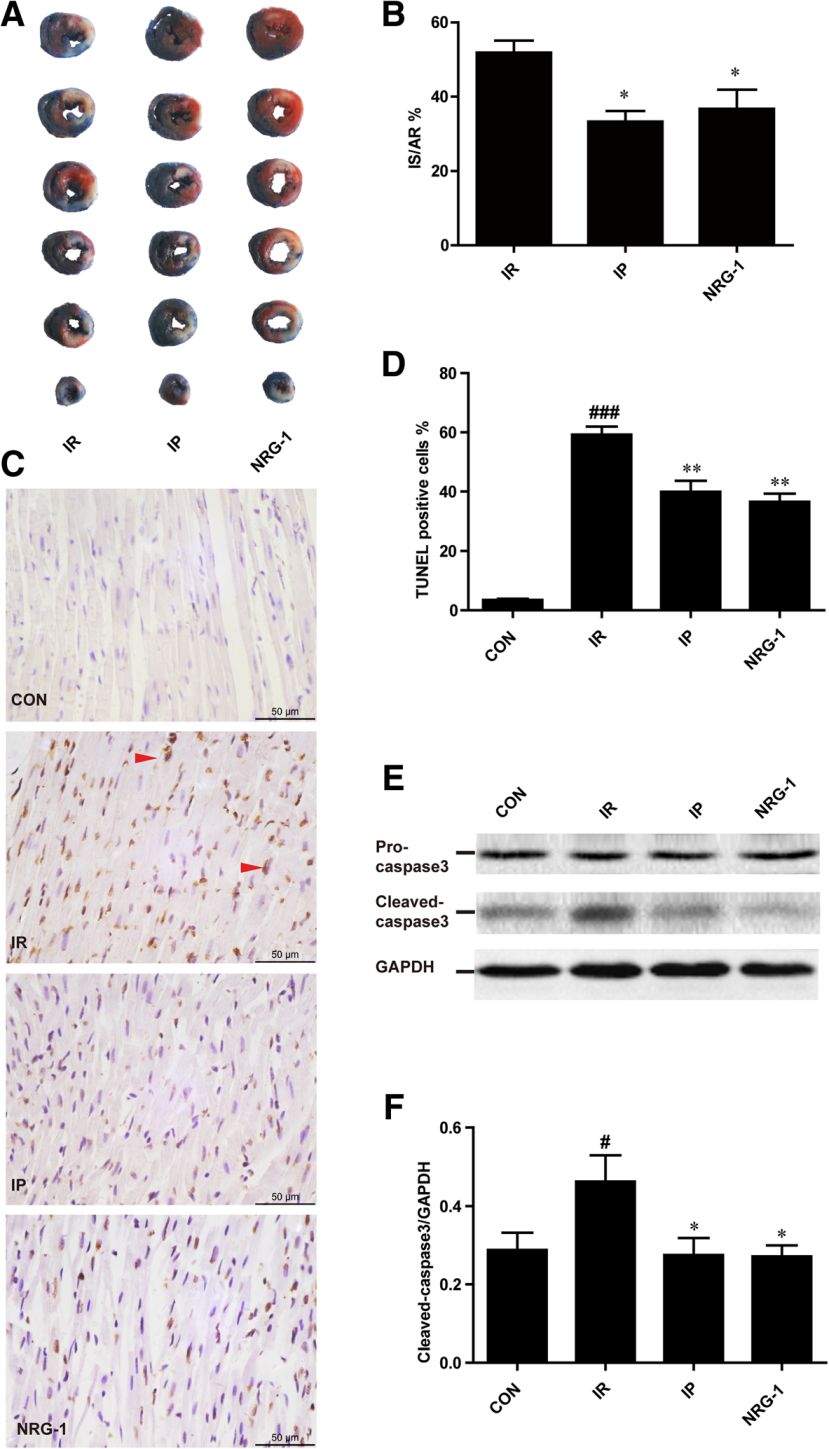
Protective effects of both IP and NRG-1 by reducing the IS and apoptosis induced by IR in vivo*.* (**a**), Representative heart slices stained by Evans blue and TTC. Blue: non-ischaemic area; non-blue: the area at risk (AR); white: infarct size (IS). (**b**), The percentage of infarct size/area at risk (IS/AR%). (**c**), Representative myocardial apoptosis in paraffin sections of the heart at the risk area. The normal cellular nuclei were stained blue by haematoxylin; the apoptotic nuclei were stained brown by TUNEL assay. (**d**), The percentage of TUNEL-positive cells in the total cells. (**e**), Representative protein levels of pro-caspase 3 and cleaved-caspase 3 by western blotting. (**f**), Semi-quantification of cleaved-caspase 3 protein levels normalised to GAPDH. CON: control, IR: ischaemia-reperfusion, IP: ischaemic postconditioning, NRG-1: IR + NRG-1. Data are shown as the mean ± SEM (*n* = 6). **#***p* < 0.05, **###***p* < 0.001 vs. CON, *p < 0.05, **p < 0.01 vs. IR

After 24 h reperfusion in the IR rat, we compared the cardioprotective effects of IP and NRG-1. The heart slices were stained by Evans blue and TTC, distinguishing the non-ischaemic area (blue region), the area at risk (AR, non-blue region) and IS (white region) (Fig. 1a). The IS/AR% was used to evaluate the damage to the heart as previously described (Xie et al., 2015). Both IP and NRG-1 treatment effectively reduced IS to similar levels compared with the IR group (33.37 ± 2.86% and 36.82 ± 5.04% respectively, vs. 51.87 ± 3.27%, *p* < 0.05; Fig. 1b).

The apoptotic nuclei were stained brown by TUNEL assays. We found that apoptosis increased in the IR group compared to the control group as shown in Fig. 1c-d (*p* < 0.001). The increase of apoptosis induced by myocardial reperfusion injury was suppressed by both IP and NRG-1 treatment (Fig. 1d, *p* < 0.01). Pro-caspase 3 (35 kDa) and its activated fragment cleaved-caspase 3 (17 kDa), as the executor of apoptosis, were assessed by western blotting (Fig. 1e). Cleaved-caspase 3 protein levels were significantly increased in the IR group compared to the control group (Fig. 1f, *p* < 0.05) and decreased in the IP and NRG-1 groups compared with the IR group (Fig. 1f, p < 0.05). Pro-caspase 3 protein levels remained unchanged.

### Activation of NRG-1/ErbB4 by IP in vivo

**Fig. 2:**
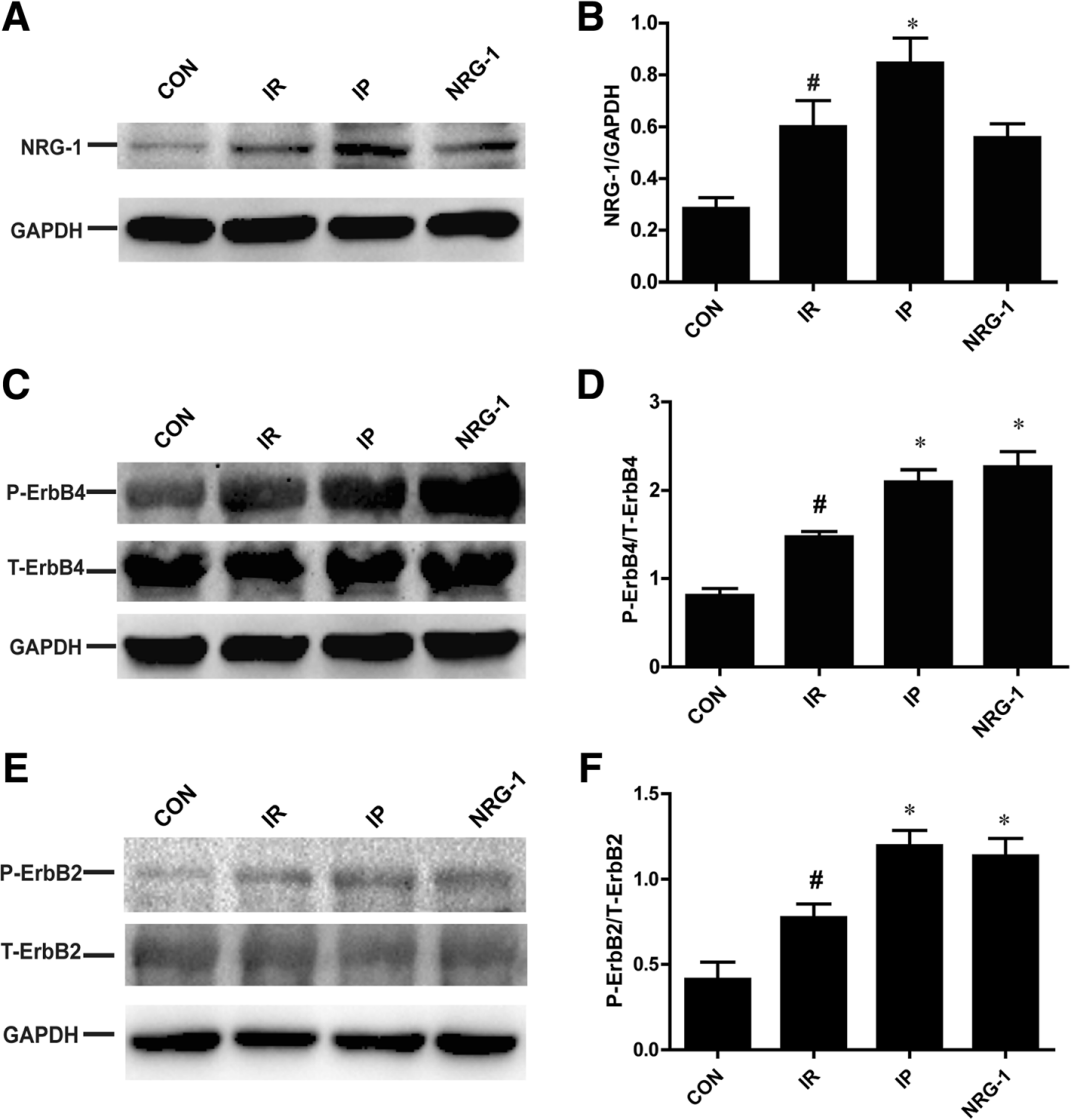
Increased NRG-1 and activation of ErbB2/4 by both IP and NRG-1 in vivo. (**a**), Representative protein levels of NRG-1. (**b**), Semi-quantification of protein levels of NRG-1. (**c**), Representative protein levels of P-ErbB4 and T-ErbB4 by western blotting. (**d**), Semi-quantification of the density ratio of P-ErbB4/T-ErbB4. (**e**), Representative protein levels of P-ErbB2 and T-ErbB2. (**f**), Semi-quantification of protein levels of P-ErbB2/T-ErbB2.These protein levels were normalised to GAPDH. CON: control, IR: ischaemia-reperfusion, IP: ischaemic postconditioning, NRG-1: IR + NRG-1. Data are shown as the mean ± SEM (*n* = 6). **#** *p* < 0.05 vs. CON, *p < 0.05 vs. IR

To clarify the relationship between IP and NRG-1, we measured the protein expression of NRG-1 (70 kDa), ErbB2 (138 kDa) and ErbB4 (185 kDa) (Fig. 2). After 24 h reperfusion, NRG-1 increased significantly in the IR group compared with control group (Fig. 2b, *p* < 0.05). These results were consistent with a previous study (Fang et al., 2010). A further increase of NRG-1 was detected in the IP group compared with the IR group (Fig. 2b, *p* < 0.05). ErbB4 is the receptor of NRG-1. Phosphorylation levels of ErbB4 were higher in the IR than in the control group, and the levels were further increased in the NRG-1 group compared to the IR group as expected (Fig. 2d, p < 0.05). Interestingly, activation of ErbB4 was also observed in the IP group (Fig. 2d, p < 0.05). Total ErbB4 levels remained unchanged in all group. ErbB2 has no direct ligand, but it can be activated by forming a heterodimer with ErbB4 which then triggers the downstream signals. In the NRG-1 and IP group, the phosphorylation of ErbB2 increased significantly compared with the IR group (Fig. 2f, *p* < 0.05).

### Activation of the RISK pathway by IP and NRG-1 in vivo

**Fig. 3:**
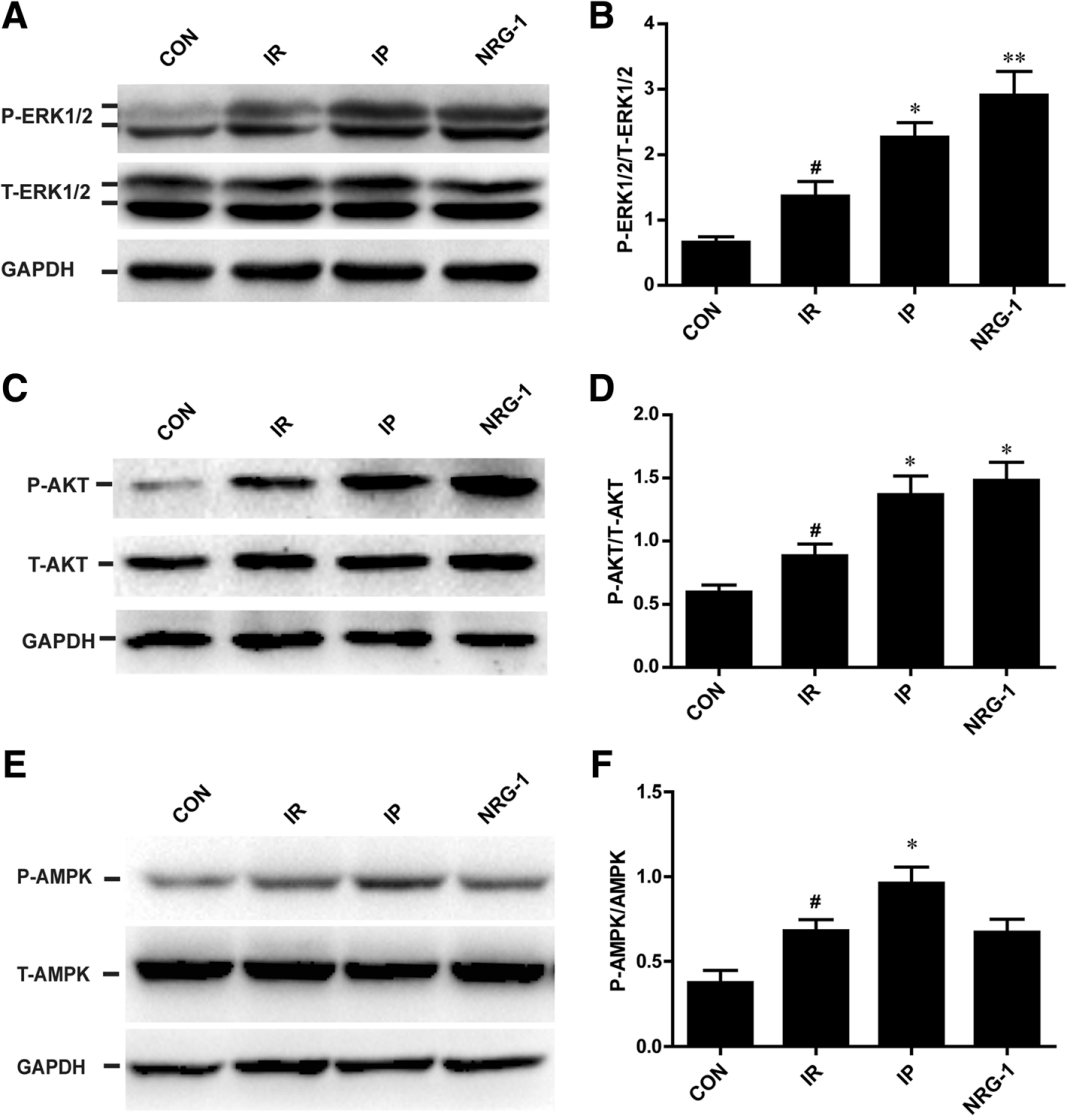
Activation of the RISK pathway by IP and NRG-1 in vivo. (**a**), Representative protein levels of P-ERK1/2 and T-ERK1/2 by western blotting. (**b**), Semi-quantification of the density ratio of P-ERK1/2/T-ERK1/2. (**c**), Representative protein levels of P-AKT and T-AKT by western blotting. (**d**), Semi-quantification of the density ratio of P-AKT/T-AKT. (**e**), Representative protein levels of P-AMPK and T-AMPK by western blotting. (**f**), Semi-quantification of the density ratio of P-AMPK/T-AMPK. These protein levels were normalised to GAPDH. CON: control, IR: ischaemia-reperfusion, IP: ischaemic postconditioning, NRG-1: IR + NRG-1. Data are shown as the mean ± SEM (n = 6). **#***p* < 0.05 vs. CON; *p < 0.05, **p < 0.01 vs. IR

**Fig. 4:**
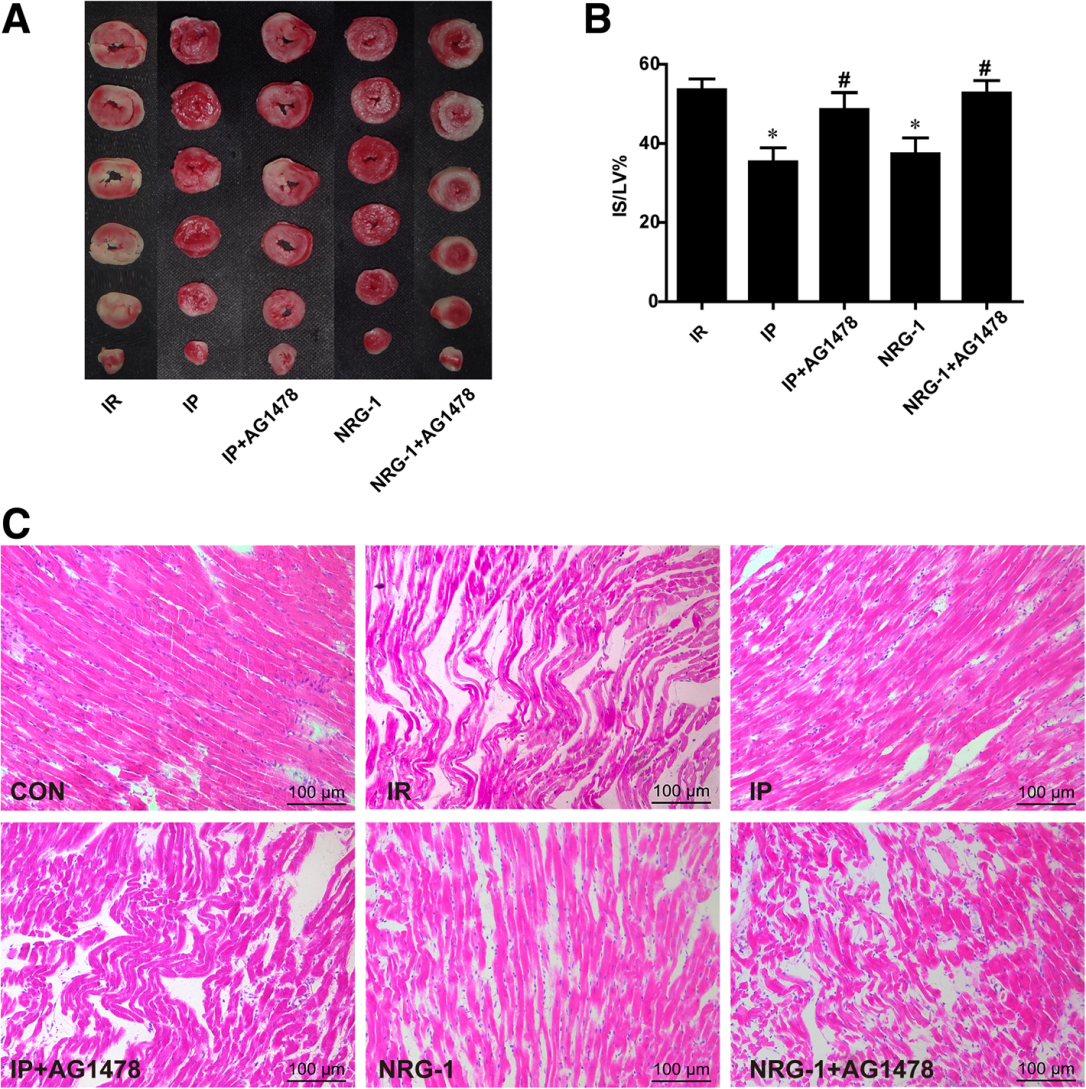
Protective effects of IP and NRG-1 were suppressed by the ErbB4 inhibitor AG1478 ex vivo*.* (**a**), Representative heart slices stained by TTC, red: the ischaemic area, white: infarct area. (**b**), the percentage of infarct size/left ventricle (IS/LV%). (**c**), Histological analysis of heart sections in Langendorff model by hematoxylin and eosin staining. CON: control, IR: ischaemia-reperfusion, IP: ischaemic postconditioning, NRG-1: IR + NRG-1. Data are shown as the mean ± SEM (*n* = 6). *p < 0.05 vs. IR; **#***p* < 0.05 vs. the same treated group without AG1478

The phosphorylation of AKT (60 kDa) and ERK1/2 (44 kDa/42 kDa) was detected by western blotting at the end of 24 h reperfusion as shown in Fig. 4. Following IP or NRG-1 treatment, conspicuous increases in phosphorylation of AKT and ERK1/2 were observed compared with the IR group (Fig. 3b, d). Moreover, there was no significant difference between the IP group and NRG-1 group. The phosphorylation of AMPK (62 kDa) has been showed to play a crucial role in the process of IP. Compared with IR group, the phosphorylation of AMPK was elevated in the IP group, but no significant increase was found with the NRG-1 treatment (Fig. 3e, f).

### The cardioprotective effects of IP were suppressed by the ErbB4 inhibitor ex vivo

To determine the role of NRG-1 in the IP process, we used the ErbB4 inhibitor AG1478 to block NRG-1 in the Langendorff isolated rat heart perfusion model. After 2 h reperfusion, IS was significantly reduced in the IP and NRG-1 groups compared with the IR group (Fig. 4a, b, *p* < 0.05). The protective effects induced by IP and NRG-1 showed no significant difference. When the ErbB4 inhibitor AG1478 was perfused to the heart, reduction of the IS by IP and NRG-1 treatment was suppressed (Fig. 4b). H&E staining was performed for histological analysis. The myocardial structure in IR group showed irregularly arranged muscle fibers; fracture, degeneration, and necrosis of muscle fibers; severe edema between cells, pyknotic nuclei, and infiltrated inflammatory cells. IP and NRG-1 treatments showed similar effects on reducing the pathological changes of myocytes. AG1478 could suppress the protective effects of IP and NRG-1 on the IR induced pathological changes (Fig. 4c).

### Phosphorylation of ErbB4 and RISK pathway were decreased by the ErbB4 inhibitor ex vivo

**Fig. 5:**
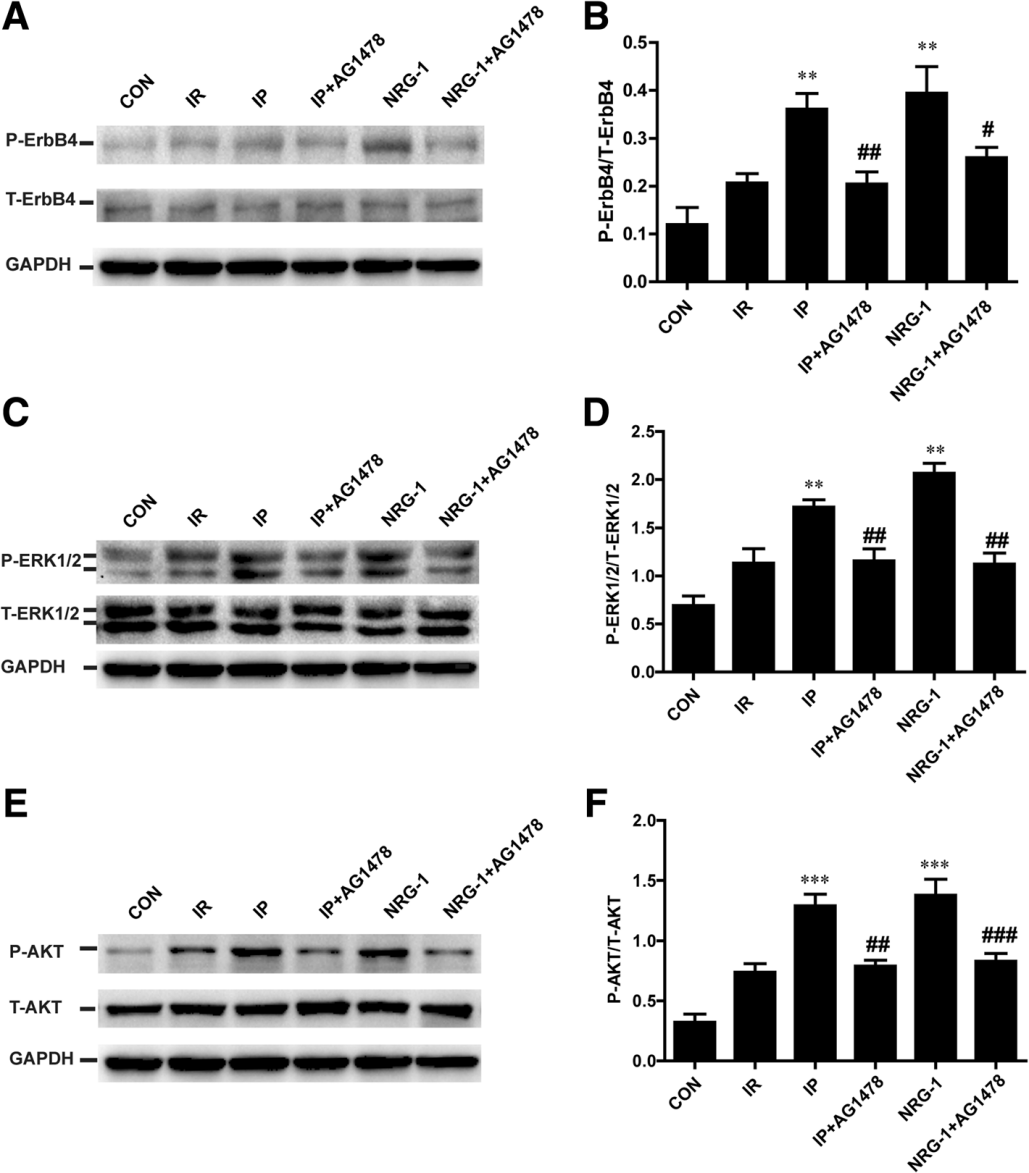
The phosphorylation of ErbB4 and the RISK pathway inhibited by AG1478 ex vivo. (**a**), Representative protein levels of P-ErbB4 and T-ErbB4 by western blotting. (**b**), Semi-quantification of the density ratio of P-ErbB4/T-ErbB4. (**c**), Representative protein levels of P-ERK1/2 and T-ERK1/2 by western blotting. (**d**), Semi-quantification of the density ratio of P-ERK1/2/T-ERK1/2. (**e**), Representative protein levels of P-AKT and T-AKT by western blotting. (**f**), Semi-quantification of the density ratio of P-AKT/T-AKT. These protein levels were normalised to GAPDH. CON: control, IR: ischaemia-reperfusion, IP: ischaemic postconditioning, NRG-1: IR + NRG-1. Data are shown as the mean ± SEM (*n* = 6). *p < 0.05, **p < 0.01, ***p < 0.001 vs. IR; **#***p* < 0.05, **##***p* < 0.01, **###** *p* < 0.001 vs. the same treated group without AG1478

To clarify the potential role of ErbB4 in IP, we detected the phosphorylation of ErbB4. The phosphorylation of ErbB4 induced by IP and NRG-1 was significantly suppressed by AG1478 in isolated heart (Fig. 5a). To determine whether ErbB4 plays a role in IP-induced phosphorylation of ERK1/2 and AKT, we reperfused the heart with AG1478 in a Langendorff apparatus. After 20 min reperfusion, the phosphorylation of AKT and ERK1/2 increased substantially after IP and NRG-1 treatment compared with the IR group, consistent with the in vivo study (Fig. 5c-f). Following pretreatment of AG1478, the phosphorylation of ERK1/2 and AKT induced by IP or NRG-1 was reduced considerably compared to the non-AG1478 treated groups (Fig. 5c-f).

### The cardioprotective effects of IP and NRG-1 were suppressed by PD98059 and LY294002 ex vivo

**Fig. 6:**
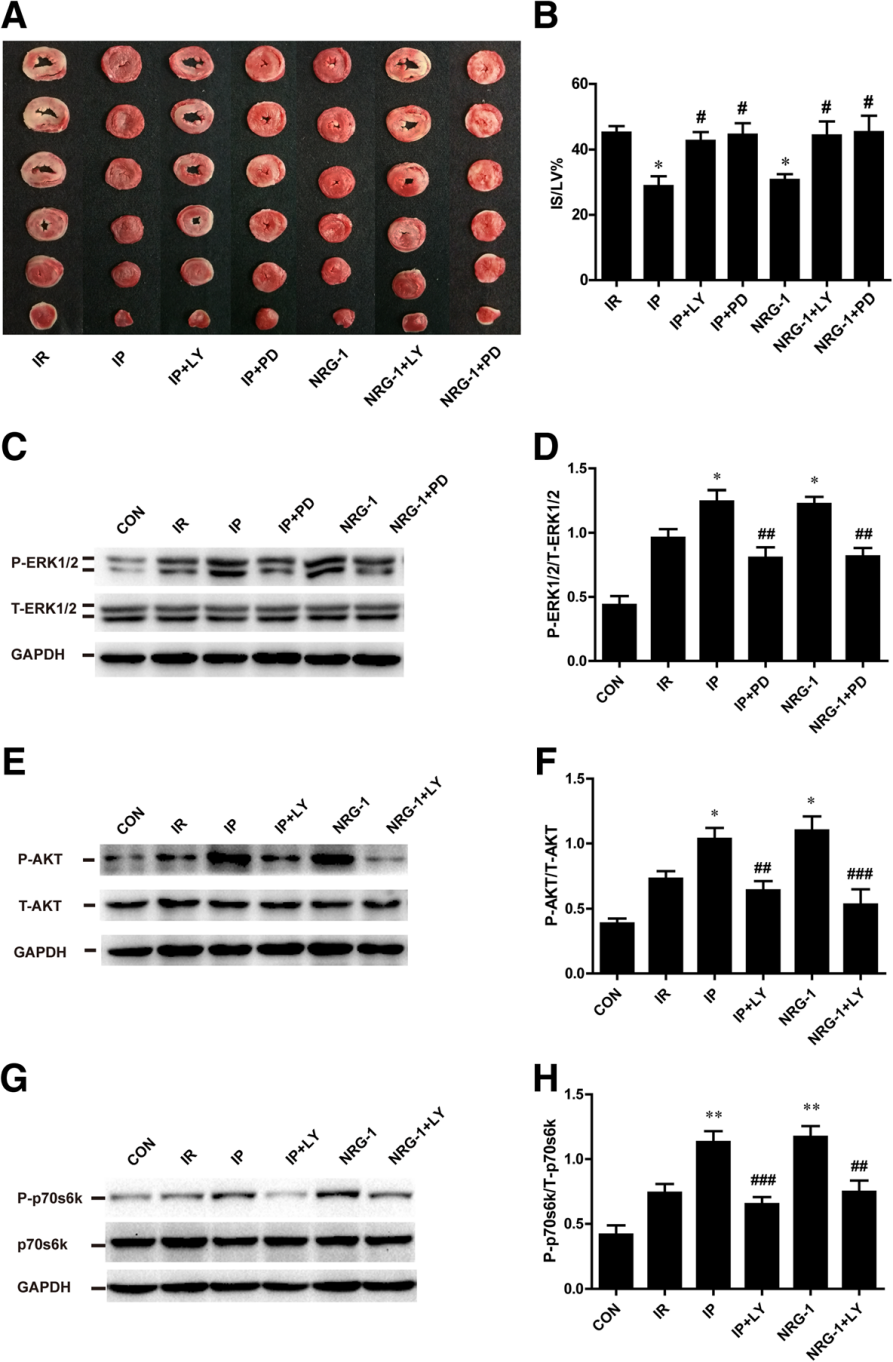
Protective effects of IP and NRG-1 abolished by PD980509 and LY294002 ex vivo. (**a**), Representative heart slices stained by TTC, red: the ischaemic area, white: infarct area. (**b**), The percentage of infarct size/left ventricle (IS/LV%). (**c**), Representative protein levels of P-ERK1/2 and T-ERK1/2 by western blotting. (**d**), Semi-quantification of the density ratio of P-ERK1/2/T-ERK1/2. (**e**), Representative protein levels of P-AKT and T-AKT by western blotting. (**f**), Semi-quantification of the density ratio of P-AKT/T-AKT. (**g**), Representative protein levels of P-p70s6k and T-p70s6k by western blotting. (**h**), Semi-quantification of the density ratio of P-p70s6k/T-p70s6k.These protein levels were normalised to GAPDH. CON: control, IR: ischaemia-reperfusion, IP: ischaemic postconditioning, NRG-1: IR + NRG-1, PD: PD98059, LY: LY294002. Data are shown as the mean ± SEM (*n* = 6). *p < 0.05, **p < 0.01 vs. IR; **#***p* < 0.05, **##***p* < 0.01, **###***p* < 0.001 vs. the same treated group without inhibitor

To determine whether the cardioprotective effects induced by IP and NRG-1depend on RISK pathway activation, we used LY294002 (LY, a PI3K inhibitor) and PD98059 (PD, a MEK inhibitor) to block the phosphorylation of AKT and ERK1/2, respectively, in the Langendorff isolated rat heart perfusion model. After 20 min reperfusion, treatment with LY or PD considerably inhibited the phosphorylation of AKT and ERK1/2 induced by NRG-1 and IP (Fig. 6c-f). In addition to suppression of the RISK pathway, the reduction of IS induced by IP and NRG-1 was reversed by LY and PD (Fig. 6a-b). For downstream signals, we measured the phosphorylation of p70S6K which has been shown to be activated by AKT. P-p70S6K was up regulated in the IP and NRG-1 group than in the IR group. Treatment with LY substantially reduced P-p70S6K induced by IP and NRG-1(Fig. 6g-h).

## Discussion

The present study demonstrated that NRG-1 shows similar cardioprotective effect to those of IP via activation of the NRG-1/ErbB pathway and downstream PI3K-AKT and ERK1/2 signalling, which is termed the RISK pathway. To our knowledge, this is the first study that used a head-to-head comparison of NRG-1 and IP to demonstrate that the NRG-1/ErbB signalling pathway mediates the cardioprotection of IP and the potential use of NRG-1 as a promising agent of pharmacological postconditioning.

The NRG-1/ErbB pathway is considered a compensatory protective mechanism of cardiac injury (Odiete et al., 2012). A recent study indicated that preconditioning with NRG-1 protected the heart from reperfusion injury in an IR rat model (Fang et al., 2010). However, preconditioning with injection of NRG-1 for 20 min before long-term ischaemia is difficult to perform in humans. Pharmacological postconditioning may be an effective and more applicable therapy. Our study found that postconditioning with injection of NRG-1 simultaneously with reperfusion could reduce IS and apoptosis in the IR rat model in vivo and the Langendorff model. This protective effect of NRG-1 was also confirmed by two studies in mice IR model (Ebner et al., 2015; Ebner et al., 2013). Our results revealed an increase of NRG-1 induced by IR in vivo (Fig. 2), which was described in a previous study indicating the release of NRG-1 from microvascular endothelial cells induced by hypoxia-reoxygenation (Hedhli et al., 2011). Endogenous NRG-1 was insufficient to protect against myocardial reperfusion injury (Fig. 1), but injection of exogenous NRG-1 resulted in increased activation of downstream signalling pathways compared with that of the IR group (Fig. 3).

The downstream signals of NRG-1 are complicated. After binding with NRG-1, the receptor ErbB4 is phosphorylated by the formation of dimers, and the downstream RISK signalling pathway, including AKT and ERK1/2, is activated. The AKT and ERK1/2 pathway may mediate the effect of NRG-1 signalling on the survival of cardiomyocytes (Odiete et al., 2012). Preconditioning with NRG-1 was found to protect cardiomyocyte from apoptosis by activation of AKT in a rat IR model (Fang et al., 2010). In the present study, we confirmed that postconditioning with NRG-1 could also stimulate the RISK pathway in IR rats in vivo (Fig. 3) and the in vitro Langendorff model (Fig. 5). and protected cardiomyocytes from apoptosis in vivo (Fig. 1).

Apoptosis plays important roles in myocardial reperfusion injury, and caspase 3 inhibitors could effectively reduce this injury (Hausenloy & Yellon, 2004). IP has been shown to significantly reduce the number of TUNEL-positive cells (Kin et al., 2008) and suppress the activity of caspase 3 (Tian et al., 2011). The anti-apoptotic effects of NRG-1 on cardiomyocytes were identified in many studies and were assessed on apoptosis induced by H_2_O_2_ (Xu et al., 2014), anthracycline (Fukazawa, 2003) and serum deprivation (Kuramochi et al., 2004). Thus, we explored the anti-apoptotic effect of NRG-1 in an IR rat model. Following treatment with NRG-1, TUNEL-positive cells and cleaved-caspase 3 expression were both reduced significantly compared with those of the IR group, which is similar to the effects of IP. We suggest that NRG-1 might be a promising pharmacological postconditioning agent that significantly suppresses apoptosis induced by IR. The mechanisms of reperfusion injury were complicated, and whether the cardioprotective effect of NRG-1 exclusively relies on the anti-apoptotic effect remains to be evaluated in future studies.

The cardioprotective effect of IP is well established (Heusch, 2015). IP plays an important role in reduction of oxidative stress, inflammation and apoptosis by salvage kinase pathways, including AKT (Tsang et al., 2004), ERK1/2 (Yang et al., 2004), AMPK (Hao et al., 2017), PKC and PKG (Ovize et al., 2010). No activation of AMPK in NRG-1 group suggested AMPK was not involved in the protective effect of NRG-1 (Fig. 3e-f). In this study, we found that NRG-1 and IP had similar anti-apoptotic effects by activation of the RISK pathway in the IR rat model. Increased phosphorylation of ErbB4 was detected in the NRG-1 group compared with the IR group in vivo as expected (Fig. 2). Notably, the activation of ErbB4 was also found in the IP group (Fig. 2). Therefore, we detected the protein levels of NRG-1, the ligand of ErbB4. Our data showed the IP could increase NRG-1 protein expression in the in vivo study. These results suggested that the cardioprotective effect of IP is mediated by the NRG-1/ErbB4 pathway. The shedding of NRG-1, which is regulated by a disintegrin and metalloprotease (ADAM)17/19 (Zhang et al., 2015), may also be a critical part of IP based on a study demonstrating the involvement of ADAM17 in ischaemic preconditioning (Ichikawa et al., 2004). The detailed mechanisms of NRG-1/ErbB4 pathway activation stimulated by postconditioning should be clarified by further investigations.

In the Langendorff experiments, AG1478 could effectively block the activation of NRG-1/ErbB4 pathway after IP treatment, and diminish the activation of the RISK pathway (Fig. 5). These findings suggested that the NRG-1/ErbB4 signalling pathway was involved in IP. AG1478 is not a specific inhibitor of ErbB4, it could also inhibit the activation of EGFR (ErbB1). A previous study showed no activation of EGFR after the incubation of NRG-1 in neonatal rat ventricle myocyte (NRVM) (Fukazawa, 2003). To further clarify the potential role of ErbB4 in IP, we examined the phosphorylation of ErbB4. We found the phosphorylation of ErbB4 was increased by IP and suppressed by AG1478 in isolated heart treated with IP (Fig. 5b). All of these data showed activation of NRG-1/ErbB4 pathway was involved in the protective effects of IP. In isolated cultured cardiomyocytes lacking NRG-1 expression, postconditioning exhibited protective effects against IR injury as well (Sun et al., 2005). NRG-1-independent ErbB4 activation might be involved in protective mechanisms of IP (Forrester et al., 2016). As a mechanical stimulus, IP may be associated with many mechanical and chemical signals. The mechanisms of the NRG-1/ErbB4 signalling pathway activated by IP should be further explored.

To assess the downstream signals, we used LY294002 and PD98059 to block RISK pathway activation and found that the cardioprotective effects induced by NRG-1 and IP were substantially decreased (Fig. 6c-f). Numerous studies have shown that LY can effectively inhibit the effects of NRG-1 and IP by preventing the phosphorylation of AKT, which is consistent with a present study (Tsang et al., 2004; Ebner et al., 2015). p70S6K could be activated by AKT and contributed to the cardioprotective effects (Tsang et al., 2004). Higher level of P-p70S6K was detected in IP and NRG-1 groups than IR group. This result indicated AKT/p70S6K pathway played important role in protective effect of NRG-1 against myocardial reperfusion injury. PD was reported to block the effects of IP by inhibiting the phosphorylation of ERK1/2 (Darling et al., 2005); however, whether PD can block the effects of NRG-1 in a Langendorff model is unknown. In the present study, we found that PD can inhibit the phosphorylation of ERK1/2 induced by NRG-1, which impaired protective effects (Fig. 6c-d).

In this study, we used IR rat models to compare the protective effects of NRG-1 and IP. We know difference between species could lead to different effects of the same drugs (Heusch, 2017). Protective effect of NRG-1 by activation of RISK pathway in patients suffering from myocardial IR injury remained unknown. Whether NRG-1 activated other signalling pathways besides RISK need more investigation. It is difficult to translate the findings in healthy, young animals with acute coronary occlusion/reperfusion to patients of older age, with a variety of co-morbidities and co-medications, suffering from different scenarios of myocardial IR injury. Dosing and timing studies also very important to evaluate the pharmacological effect of NRG-1. Therefore, we need more experiments in different animal models and even larger clinical trials with dosing and timing studies to clarify the protective effect of NRG-1 in patients suffering from myocardial IR injury.

## Conclusion

In conclusion, both NRG-1 and IP have cardioprotective effects by reducing IS and apoptosis through ErbB4-dependent activation of the RISK pathway in a rat myocardial reperfusion injury model. NRG-1 might be a potential pharmacological postconditioning agent for cardioprotection against reperfusion injury.

## Abbreviations

ADAM: A disintegrin and metalloprotease
AMI: Acute myocardial infarction
AR: the area at risk
CON: Control
EGF: Epidermal growth factor
ErbB4: Erythroblastic leukaemia viral oncogene homolog 4
ERK1/2: Extracellular signal-regulated kinase 1/2
H&E: Hematoxylin/eosin
IP: Ischaemic postconditioning
IR: Ischaemia-reperfusion
IS: Infarct size
K-H: the Krebs-Henseleit
LAD: the left anterior descending coronary artery
LV: the left ventricle
LY: LY294002
MEK: a mitogen-activated protein/extracellular signal regulated kinase
NRG-1: Neuregulin-1
NRVM: Neonatal rat ventricle myocyte
PD: PD98059
PI3K: Phosphatidylinositol 3-kinase
RISK: Reperfusion injury salvage kinase
TTC: Triphenyltetrazolium chloride

## References

[CR1] Bell RM, Mocanu MM, Yellon DM (2011). Retrograde heart perfusion: the Langendorff technique of isolated heart perfusion. J Mol Cell Cardiol.

[CR2] Cai MX (2016). Exercise training activates neuregulin 1/ErbB signaling and promotes cardiac repair in a rat myocardial infarction model. Life Sci.

[CR3] Darling CE (2005). Postconditioning via stuttering reperfusion limits myocardial infarct size in rabbit hearts: role of ERK1/2. Am J Physiol Heart Circ Physiol.

[CR4] Ebner B (2013). Uncoupled eNOS annihilates neuregulin-1beta-induced cardioprotection: a novel mechanism in pharmacological postconditioning in myocardial infarction. Mol Cell Biochem.

[CR5] Ebner B (2015). In situ postconditioning with neuregulin-1beta is mediated by a PI3K/Akt-dependent pathway. The Canadian journal of cardiology.

[CR6] Fang SJ (2010). Neuregulin-1 preconditioning protects the heart against ischemia/reperfusion injury through a PI3K/Akt-dependent mechanism. Chin Med J.

[CR7] Forrester SJ (2016). Epidermal growth factor receptor transactivation: mechanisms, pathophysiology, and potential therapies in the cardiovascular system. Annu Rev Pharmacol Toxicol.

[CR8] Fukazawa R (2003). Neuregulin-1 protects ventricular myocytes from anthracycline-induced apoptosis via erbB4-dependent activation of PI3-kinase/Akt. J Mol Cell Cardiol.

[CR9] Gao R (2010). A phase II, randomized, double-blind, multicenter, based on standard therapy, placebo-controlled study of the efficacy and safety of recombinant human neuregulin-1 in patients with chronic heart failure. J Am Coll Cardiol.

[CR10] Gu X (2010). Cardiac functional improvement in rats with myocardial infarction by up-regulating cardiac myosin light chain kinase with neuregulin. Cardiovasc Res.

[CR11] Hao M, et al. Myocardial ischemic Postconditioning promotes autophagy against ischemia reperfusion injury via the activation of the nNOS/AMPK/mTOR pathway. Int J Mol Sci. 2017;18 10.3390/ijms18030614 PMC5372630 28287478

[CR12] Hausenloy DJ, Yellon DM (2004). New directions for protecting the heart against ischaemia-reperfusion injury: targeting the reperfusion injury salvage kinase (RISK)-pathway. Cardiovasc Res.

[CR13] Hedhli N (2011). Endothelium-derived neuregulin protects the heart against ischemic injury. Circulation.

[CR14] Heusch G (2015). Molecular basis of cardioprotection: signal transduction in ischemic pre-, post-, and remote conditioning. Circ Res.

[CR15] Heusch G (2017). Critical issues for the translation of Cardioprotection. Circ Res.

[CR16] Ibanez B, Heusch G, Ovize M, Van de Werf F (2015). Evolving therapies for myocardial ischemia/reperfusion injury. J Am Coll Cardiol.

[CR17] Ichikawa Y (2004). The role of ADAM protease in the tyrosine kinase-mediated trigger mechanism of ischemic preconditioning. Cardiovasc Res.

[CR18] Jie B (2012). Neuregulin-1 suppresses cardiomyocyte apoptosis by activating PI3K/Akt and inhibiting mitochondrial permeability transition pore. Mol Cell Biochem.

[CR19] Kin H (2008). Inhibition of myocardial apoptosis by postconditioning is associated with attenuation of oxidative stress-mediated nuclear factor-kappa B translocation and TNF alpha release. Shock.

[CR20] King AL (2014). Hydrogen sulfide cytoprotective signaling is endothelial nitric oxide synthase-nitric oxide dependent. Proc Natl Acad Sci U S A.

[CR21] Kleinbongard P, Heusch G (2015). Extracellular signalling molecules in the ischaemic/reperfused heart - druggable and translatable for cardioprotection?. Br J Pharmacol.

[CR22] Kuramochi Y (2004). Cardiac endothelial cells regulate reactive oxygen species-induced cardiomyocyte apoptosis through neuregulin-1beta/erbB4 signaling. J Biol Chem.

[CR23] Liu FF (2005). Heterozygous knockout of neuregulin-1 gene in mice exacerbates doxorubicin-induced heart failure. Am J Physiol Heart Circ Physiol.

[CR24] Liu L (2014). MicroRNA-15b enhances hypoxia/reoxygenation-induced apoptosis of cardiomyocytes via a mitochondrial apoptotic pathway. Apoptosis : an international journal on programmed cell death.

[CR25] Mozaffarian D (2015). Heart disease and stroke statistics--2015 update: a report fro the American Heart Association. Circulation.

[CR26] Odiete O, Hill MF, Sawyer DB (2012). Neuregulin in cardiovascular development and disease. Circ Res.

[CR27] Ovize M (2010). Postconditioning and protection from reperfusion injury: where do we stand? Position paper from the working Group of Cellular Biology of the heart of the European society of cardiology. Cardiovasc Res.

[CR28] Parodi EM, Kuhn B (2014). Signalling between microvascular endothelium and cardiomyocytes through neuregulin. Cardiovasc Res.

[CR29] Sawyer DB, Caggiano A (2011). Neuregulin-1beta for the treatment of systolic heart failure. J Mol Cell Cardiol.

[CR30] Sun HY (2005). Hypoxic postconditioning reduces cardiomyocyte loss by inhibiting ROS generation and intracellular Ca2+ overload. Am J Physiol Heart Circ Physiol.

[CR31] Tamareille S (2009). Myocardial reperfusion injury management: erythropoietin compared with postconditioning. Am J Phys Heart Circ Phys.

[CR32] Tian Y (2011). Postconditioning inhibits myocardial apoptosis during prolonged reperfusion via a JAK2-STAT3-Bcl-2 pathway. J Biomed Sci.

[CR33] Tsang A, Hausenloy DJ, Mocanu MM, Yellon DM (2004). Postconditioning: a form of "modified reperfusion" protects the myocardium by activating the phosphatidylinositol 3-kinase-Akt pathway. Circ Res.

[CR34] Xie L (2015). Depletion of PHD3 protects heart from ischemia/reperfusion injury by inhibiting cardiomyocyte apoptosis. J Mol Cell Cardiol.

[CR35] Xu M (2014). Neuregulin-1 protects myocardial cells against H2 O2 -induced apoptosis by regulating endoplasmic reticulum stress. Cell Biochem Funct.

[CR36] Xu P (2015). Diet rich in docosahexaenoic acid/Eicosapentaenoic acid robustly ameliorates hepatic steatosis and insulin resistance in seipin deficient lipodystrophy mice. Nutrition & metabolism.

[CR37] Yang X-M (2004). Multiple, brief coronary occlusions during early reperfusion protect rabbit hearts by targeting cell signaling pathways. J Am Coll Cardiol.

[CR38] Zhang P, Shen M, Fernandez-Patron C, Kassiri Z. ADAMs family and relatives in cardiovascular physiology and pathology. J Mol Cell Cardiol. 2015; 10.1016/j.yjmcc.2015.10.031 26522853

